# Identifying Discrete States of a Biological System Using a Novel Step Detection Algorithm

**DOI:** 10.1371/journal.pone.0045896

**Published:** 2012-11-07

**Authors:** Jan Opfer, Kay-Eberhard Gottschalk

**Affiliations:** Institut für Experimentelle Physik, Universität Ulm, Ulm, Germany; Griffith University, Australia

## Abstract

Identification of discrete states is a common task when studying biological systems on microscopic scales. Here, we present a novel step detection algorithm that is ideally suited to locate steplike features separating adjacent plateaus, even if they are smooth and hidden by noise. It can be adjusted to detect very low or narrow steps that cannot be recognized by conventional methods. We demonstrate the applicability of the technique on various experimental data and show strong evidence of sub-10-pN steps in atomic force spectroscopy measurements performed with living lymphocytes.

## Introduction

Experimental data obtained by observing a biological system at microscopic scales often reflects multiple discrete states, e.g. the disruption of intermolecular bonds [1], [2], the unfolding of proteins [3], [4], or the stepwise movement of a molecular motor [5], [6], [7]. The study of such phenomena requires highly sensitive single-molecule techniques like optical/magnetic tweezers or atomic force microscopy. They are common tools to examine the kinetics of molecular bonds or enzymatic activity [8], because their spatial and force resolution is sufficient to analyze events on a nm and pN scale, respectively. In contrast to ensemble measurements, they allow revealing the mechanical properties of individual proteins and resolving conformational changes. For example, observation of the unbinding of membrane tubes pulled from living cells would not be possible without single-molecule techniques. However, the measured signal is impaired by thermal fluctuations, electronic noise and vibrations, as the disturbances are generally of the same order of magnitude. To analyze such data, an automated method to identify the steps marking the transitions between the discrete states of the investigated system is necessary. In a comparison of existing detection algorithms, an iterative fit procedure (“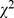 method”) proposed by Kerssemakers et al. [9] combined with a moving average filter showed the best overall performance [10]. The window size of the mean filter can be optimized for the types of steps to be recognized.

It is obvious that any information about the steps, such as their average width or signal-to-noise ratio (SNR), i.e. the ratio between height and the standard deviation of the noise, can be used to increase the probability of successful detection. In practical applications, these properties are often very similar for all steps, and their approximate heights and widths are usually known. The noise level can generally be determined from the measured data. Here, it is demonstrated that significantly higher detection rates can be obtained by a novel moving step fit (MSF) algorithm, which makes use of this information. In contrast to other methods optimized to identify changes between a small number of identical states [11], MSF is intended to reveal transitions between arbitrary discrete states. It allows the identification of low steps hidden in experimental data, which have been unrecognized before due to very low SNRs. By adjusting its fit window size, steps can be detected within two extreme cases: low and wide steps with heights far below the noise amplitude, as well as higher, but very narrow steps separated by only a few data points.

## Materials and Methods

### 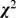 method

In contrast to the original approach by Kerssemakers et al. [9], a windowed mean filter replacing each data point in the middle of 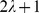 consecutive points with their average value was applied to the noisy data beforehand, because that has been shown to increase the detection rate [10]. Then, a single step is fitted to every possible position of the time trace, and the data is partitioned at the location corresponding to the smallest 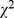 sum. The procedure is repeated iteratively with both resulting parts until the 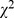 sum has been determined for every data point. Values below a threshold correspond to possible step positions. The original Matlab implementation provided by the authors [9] was used after adding the mean filter (The Mathworks, Natick, MA).

### MSF algorithm

Initially, the noisy data 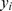 sampled at time or space intervals 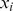 is pre-processed by convolution with a Gaussian kernel [12] with standard deviation 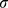. Thereby, both the source signal and the noise are smoothed, but continuous parts, such as plateaus between steps, are preserved. Then, a step of height 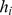 is fitted at position 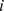 in the middle of a moving window of size 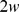 (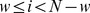). Here, a piecewise linear fit function
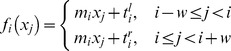
(1)with uniform slope, but different constant terms was used. A second, continuous function
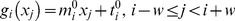
(2)is fitted to the whole window. In both cases, the global optima of the free parameters are obtained analytically:
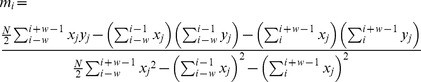
(3)

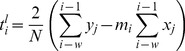
(4)

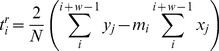
(5)

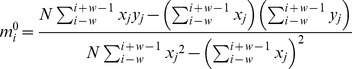
(6)

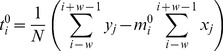
(7)


At each position the residual sum of squares (RSS) is calculated for 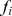 and 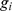. The term
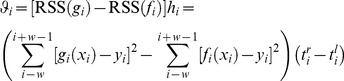
(8)only takes on high values if the step function 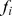 fits better than the continuous function 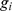. Multiplication by the step height 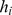 is optional and assures that large steps are more likely to be detected. Therefore, 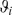 is an indicator for the probability of 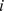 to be a potential step position. Consequently, local maxima exceeding a threshold significantly above the statistical fluctuations of 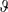 can be regarded as steps. If the number of steps, 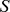, is known a priori, the 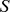 highest local maxima define the step positions instead.

### Generation of the test signals

Different types of test signals were used to evaluate the 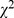 and MSF method (see table 1 and fig. S1):

**Table 1:** Test signals used for the evaluation of the step detection algorithms.

data set	description
A	2 steps with constant plateaus separated by a variable distance superimposed by additive white Gaussian noise of variable amplitudes (100 curves for each step distance and noise level with N = 4200 data points)
B	Monte Carlo simulations of force curves obtained by atomic force spectroscopy with a variable number of steps (1 to 100) at random positions superimposed by instrumental noise of variable amplitudes (100 curves for each number of steps and noise level with N = 8192 data points)
C	artificial force curves containing 4 steps at fixed positions (2, 6, 10, and 14 µm) with randomly selected heights (5, 10, 20, or 40 pN) superimposed by white or instrumental noise with a standard deviation of 10 pN (1000 curves with N = 8192 data points)

**Figure 1:**
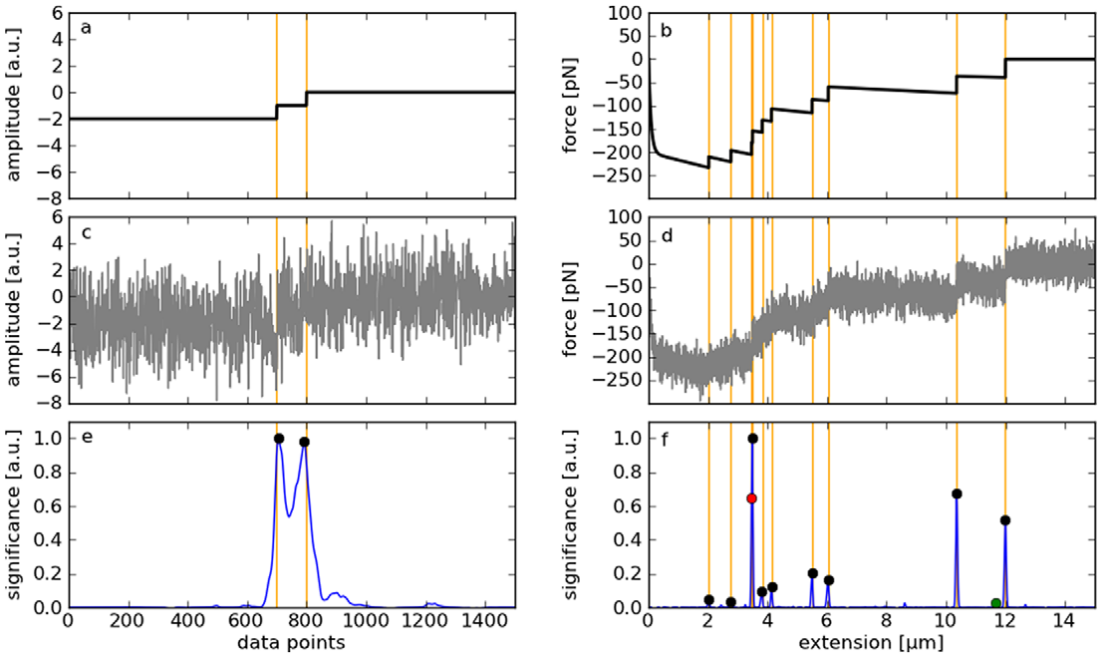
Evaluation of the MSF algorithm. a: Synthetic test signal containing two steps of height one separated by a defined distance (data set A). b: Artificial AFM spectrum generated by Monte Carlo simulations mimicking an idealized (noise-free) force-distance curve typically obtained by cell adhesion measurements (data set B). c: Clean signal superimposed by normal-distributed white noise. d: Clean signal superimposed by random AFM noise with a standard deviation of 20 pN. e, f: Indicator of possible step positions calculated from the noisy signals by the MSF algorithm (blue). Local maxima are used to identify the steps (see methods). Orange lines mark the true step positions, black dots correct detections, the green dot a false-positive, and the red dot a false-negative.

Data set A consists of curves with 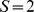 steps of height one separated by a variable distance (see example in fig. 1a).

For data set B, artificial force spectra were generated by Monte Carlo simulations (see example in fig. 1b). They contain a given number of steps, 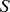, with randomly determined positions and heights. In brief, for 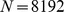 tether extensions 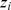 sampled at small time intervals 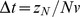, the rupture probability 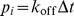 was calculated from the force-dependent off-rate [13]

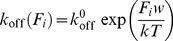
(9)using the force
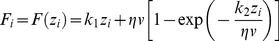
(10)exerted by a Kelvin body representing a single tether [14]. Each 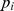 is compared with a random number 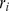 in the range 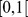 and the first occurrence of 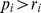 is considered a rupture event, i.e. 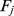 is set to zero for 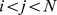. To obtain curves with multiple steps, the procedure is repeated and the forces 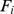 are summed up. The experiment-specific parameters were chosen to mimic real single-molecule force spectroscopy data obtained with biological cells [14], [15]: 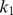 = 1.6 pN/µm, 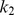 = 260 pN/µm, 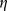 = 5.9 µNs/m, 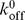 = 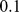 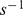, 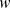 = 1.8 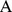, 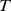 = 36°C, 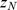 = 16.0 µm, 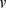 = 3.4 µm/s. As it is common practice, forces are plotted with reversed sign.

To allow for a quantification of force resolution, data set C was designed to contain steps with discrete heights – as opposed to the continuous distribution of step heights resulting from the Monte Carlo simulations. Exactly four steps with randomly chosen heights (5, 10, 20, or 40 pN) were placed at fixed positions (2, 6, 10, and 14 µm). To resemble data set B, linear plateaus with slope 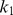 were created between the steps.

The signals of data set A were contaminated with additive white Gaussian noise. The artificial force curves of data sets B were superimposed by normal-distributed random noise with a frequency spectrum measured on a Nanowizard II atomic force microscope (JPK, Berlin, Germany), and both types of noise were applied to data set C.

White Gaussian noise was created by a Box-Muller transform [16] of uniformly distributed random numbers generated by the Mersenne Twister algorithm [17]. To reproduce unlimited amounts of the instrumental noise, force signals free of sample-specific effects were recorded with the atomic force microscope and de-trended by subtraction of a linear baseline. Their average spectral distribution was calculated by fast Fourier transform using a block size of 2^8^ and a Hann window to reduce spectral leakage. Noise signals of various amplitudes were generated by inverse Fourier transforms of this spectrum with uniformly distributed random phase shifts.

### Evaluation of the step detection algorithms

To evaluate the step detection performance of the algorithms described above, their efficiencies are quantified to localize the steps contained in the noisy test signals. The problem of finding an estimate for the true number of steps, 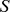, is excluded from the analysis, because a wrong number would affect the results, so that an unbiased comparison of the actual detection performance would not be possible.

Both detection methods generate a measure for the estimated probability (“significance”) of any data point to be a step. The 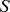 highest local maxima of this indicator define the (potentially false) identified step positions (fig. 1e and f). A detected step is rated a false-positive if the deviation from its true position is greater than ±4 data points, and a true step is rated a false-negative if the deviation from its detected position is greater than ±4 data points. Since 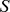 step candidates are tested, each missed true step implies the false detection of a non-existent step and vice versa. Hence, the numbers of false-positives and false-negatives are equal for each test signal. The numbers of false detections were recorded and the rate of successful detections was calculated according to the formula
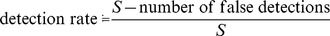
(11)


For each noise-free test signal, detection method, and noise level, the evaluation was repeated at least 100 times with distinct random noise.

The method-specific parameters were chosen to maximize detection rates for the average SNRs and widths of the steps contained in the test signals. This is only possible, because these properties are a priori known. In practical applications, optimal settings must be determined either manually or calibrated by simulated data (see section “Parameter optimization”). Data evaluation was done with Matlab and Python.

### Calculation of the step heights

Linear fits
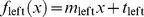
(12)

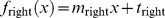
(13)were performed over up to 2048 data points to the left and to the right of the identified step positions 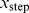, but no further than to the neighboring detected steps. The step heights were determined from the difference of the values of both fit functions at the positions of the steps:
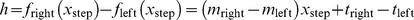
(14)


### Recording of the AFM spectra

A Nanowizard II atomic force microscope (JPK, Berlin, Germany) was deployed to measure force-distance curves of β_1_ integrin-deficient Jurkat A1 lymphocytes with re-substituted β_1_ integrin [18] interacting with the VLA-4 ligand VCAM-1 as described by Schmitz et al. [14]. A VCAM-1 concentration of 2 µg/ml and a constant approach/retract velocity of 3.4 µm/s were used.

## Results and Discussion

### Step detection performance

To render a quantitative evaluation of correct and false detections possible, the step positions must be a priori known. For that reason, the step detection performance of MSF is compared with the 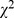 method [9] by means of synthetic test signals (fig. S1 in the Supporting Information). The 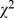 method was chosen as reference, because it has been shown to perform best among other highly efficient techniques [10].

Two different types of signals were analyzed (table 1): First, simple curves with two steps of height one separated by a variable distance were deployed (data set A, see example in fig. 1a, c) to study the influence of the SNR and of the distance between successive steps on the detection rates. The clean signals were contaminated by additive white Gaussian noise. Second, artificial force-distance curves mimicking single-molecule force spectroscopy experiments with living cells were created by Monte Carlo simulations and superimposed by the characteristic instrumental noise of a JPK Nanowizard II AFM (data set B, see example in fig. 1b, d). Such experiments are highly relevant to understand cell-surface or cell-cell adhesion and cellular force sensing [14]. Every simulated curve contains a predefined number 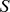 of steps at random positions. Since the 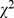 method requires a manual selection of the number of steps to be detected, a comparison how accurately 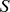 can be determined is not possible. Thus, the number of steps was assumed to be known, i.e. both algorithms were configured to detect the 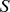 most significant steps. As a consequence, the numbers of false-positives and false-negatives are equal and need not be compared separately. For practical applications, an automatic selection of 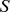 might be required. Therefore, the MSF algorithm can also be deployed with a given detection sensitivity, i.e. any steps with a significance exceeding a given threshold are detected (see methods).

**Figure 2:**
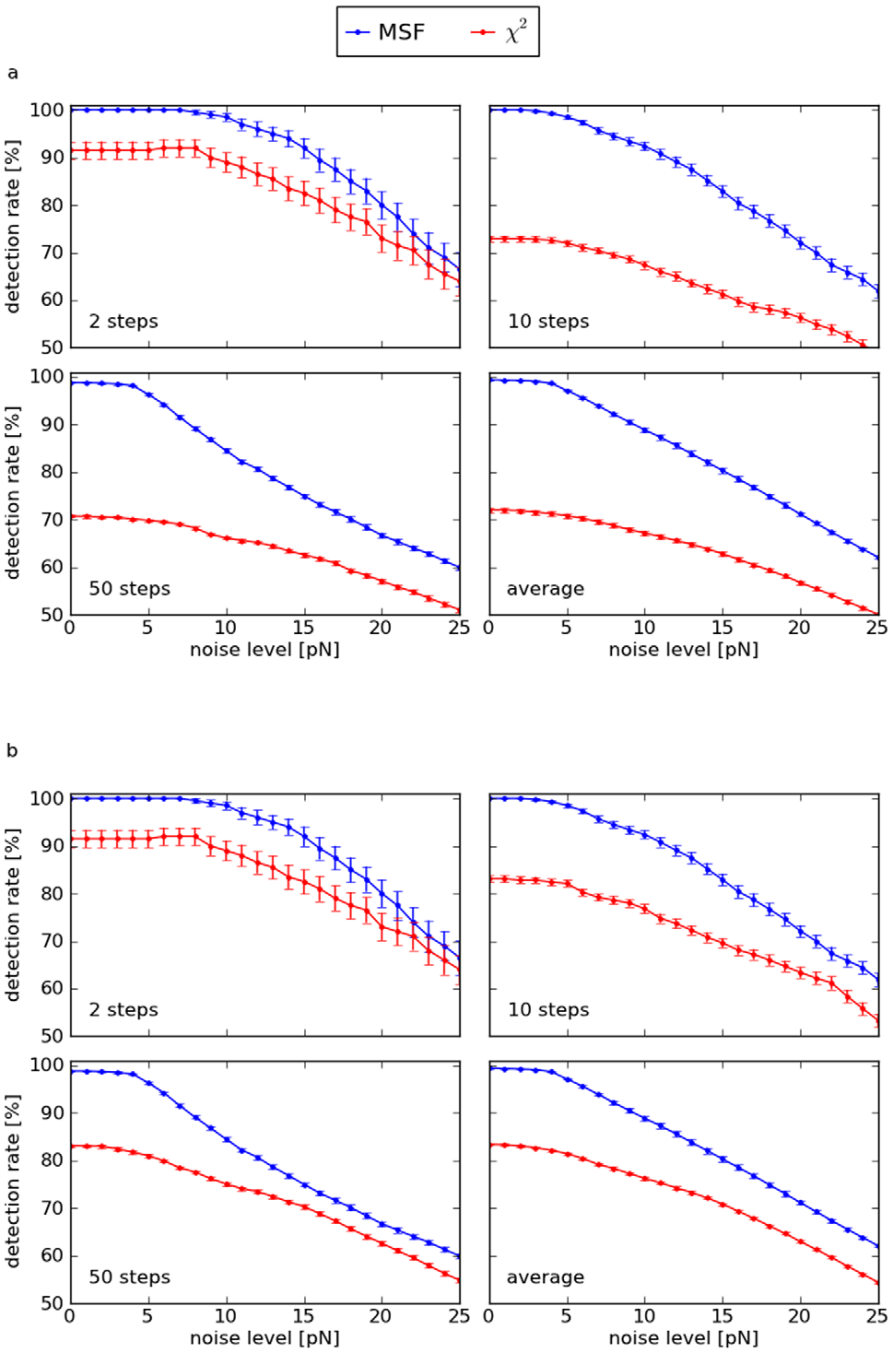
Detection rates vs. noise amplitudes of the MSF and the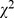 method applied to synthetic test signals. Every marker represents the average rate over 100 curves with distinct random noise, with error bars indicating the standard errors. a: Two steps of height one separated by a variable distance (data set A, see example in fig. 1c). The last plot shows the average detection rates over 33 step distances between 40 and 1000 data points. Detection parameters were optimized for the constant SNRs and distances of the steps. b: Artificial force curves generated by Monte Carlo simulations with varied number of steps (data set B, see example in fig. 1d). Parameter optimization was performed for each noise level and number of steps. The last plot shows the average detection rates over the range of 2, 3, …, 50 steps.

The efficiencies of both step detection methods depend not only on the signal and noise characteristics, but also on the choice of parameters: The 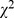 algorithm can be optimized by varying the window size 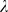 of the moving average filter and MSF by varying the width of the smoothing kernel 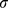 and the half window size 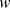 (see methods). As optimal parameters in the sense of maximum detection rates depend on the SNRs and widths of the steps in a complex way, they were determined numerically by evaluating test signals with pre-defined characteristics and a priori known step positions (see section “parameter optimization” below). In doing so, four scenarios were considered, each for data set A and B: First, the average SNR and either the average width (for set A) or the number of the steps (for set B) were assumed to be known and fairly constant, i.e. both facts were used for parameter selection (fig. 2a and b). In the second case, very different step widths (or numbers of steps) can occur, i.e. parameters were optimized for each noise level (i.e. the constant SNR for data set A and an average SNR for data set B) and for a broad range of step distances (fig. S2a and b). Third, parameters were chosen to yield highest detection rates for an approximately constant SNR and variable step distances (fig. S2c and d). If neither the SNRs nor the widths of the steps can be narrowed down, optimization must be performed for arbitrary step characteristics within a reasonable range (fig. S2e and f), resulting in constant parameters for all test signals (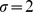, 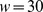, and 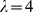).

Generally, attainable detection rates depend on the step characteristics and on the type of noise. If either the SNRs or the widths of the steps are similar, detection rates can be highly improved by MSF in comparison to the 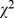 method. MSF is particularly effective for curves with many or narrow steps. Even if both the heights and the widths of the steps vary, it yields higher detection rates in many cases for both types of test signals and shows the best overall performance with about 20% higher detection rates than the 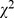 algorithm for data set A and about 30% for data set B (table 2). The Kerssemakers method combined with the moving average filter works well with curves containing few steps. It is less effective in general, because the 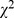 sum calculated at a potential step position is increased by other steps, making it a less sensitive indicator. Only curves with at least 2 steps could be included in the analysis, as the algorithm fails if the number of steps to be detected is set to 1. Further, the decay in the beginning of the artificial force curves of data set B impairs the method and results in some false-positive detections.

**Table 2:** Total average detection rates for the data shown in fig. 2.

	data set A		data set B	
step detection method	absolute detection rate [%]	relative detection rate [%]	absolute detection rate [%]	relative detection rate [%]
MSF	65.4	119.9	83.6	131.3
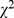	54.5	100.0	63.7	100.0

Filter settings were individually optimized for every noise amplitude and step width (data set A)/number of steps (data set B). Relative rates are related to the results obtained by the 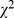 method combined with the windowed mean filter.

### Distribution of false-positives and -negatives

The real AFM measurements modeled by the simulated force curves typically show a noise level of about 10 pN and no more than 10 steps. Therefore, any further analysis of data set B is restricted to curves with 2 to 10 steps and 10 pN AFM noise. On these conditions, optimal detection rates are obtained by 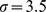, 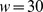, 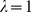. If not stated otherwise, these parameters are used in the following.

A detected/unrecognized step is rated a false-positive/false-negative if the deviation from the nearest true position is greater than ±4 data points. Otherwise it is correct by definition. Both false-positive and false-negative detections decrease with increasing step heights, as higher steps can be identified more reliably. The number of false-negatives is much lower for MSF than for the 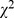 method and the number of false-positives is similar (fig. S3).

### Height resolution

To analyze real data, generally not only the step positions, but also their heights must be determined. The latter process depends on the former, and both are error-prone. The precision of the height estimation achievable with MSF and the 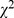 method is quantified by the example of data set C, which also models AFM force curves, but contains exactly 4 steps at 2, 6, 10, and 14 µm with discrete heights randomly chosen from 5, 10, 20, and 40 pN (see table 1 and examples in fig. S1). These modifications render it possible to determine the height resolution limit. Again, the signals were contaminated by AFM noise of a single amplitude (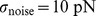) and the method-specific parameters were chosen to maximize the total average detection rates (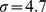, 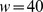, 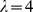). The curves consist of linear plateaus, so that the estimated step heights can be calculated from adjacent linear fits of these plateaus left and right of the steps that have been identified by the MSF algorithm (see methods).

To resolve the heights of the steps, they must be detected in the first place. MSF yields more false-positives and less false-negatives than the 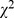 method, which does not reproduce the 5 pN peak at all (fig. S4).

**Figure 3:**
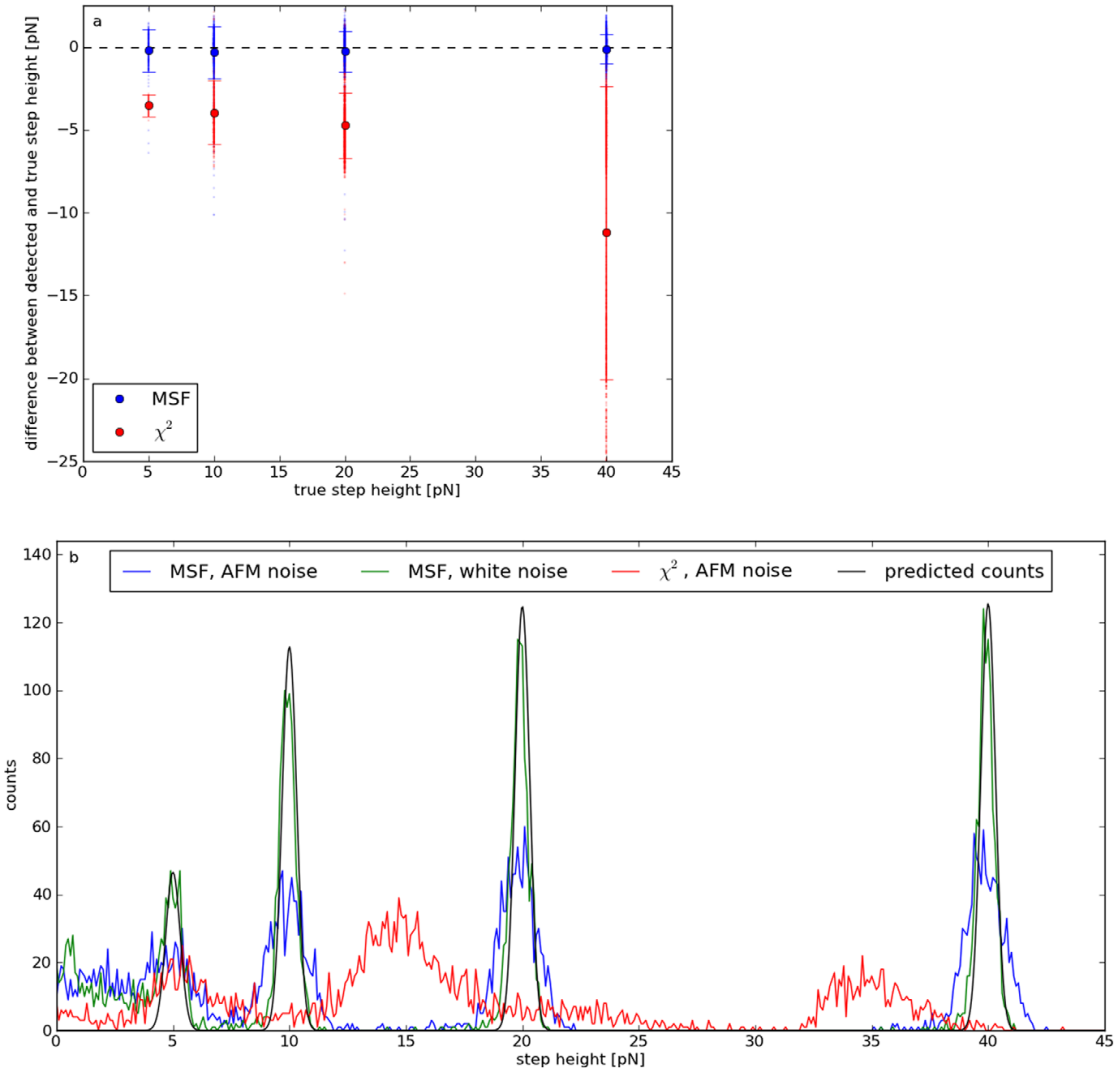
Accuracy of detected step heights. a: Mean deviations between calculated and true step heights of data set C. Only steps correctly identified by MSF (blue) or the 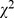 method (red) are included. The error bars indicate the standard deviations. b: Number of steps detected by MSF vs. calculated step heights for white noise (green) and AFM noise (blue). The results for the 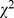 method are drawn in red and the predicted histogram for white noise in gray. Independently of the step height, individual distributions can be clearly verified if they are sufficiently far away from each other (less than 5 pN for both types of noise). The colored AFM noise results in broader peaks.

The height calculations were performed according to eq. 12–14 with 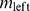 and 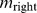 fixed to the constant slope of the plateaus (1.6 pN/µm). The total average deviation between true and estimated heights is (−0.22±1.20) pN, i.e. systematic errors (e.g. arising from false-negative detections within the fit range) are much smaller than the statistical errors resulting from the noise (fig. 3a). Step heights determined by the 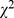 algorithm deviate from the true values by (−7.45±6.87) pN. They are considerably underestimated, because they are calculated from the difference of the mean force of the left and right edge, and not by linear fits.

As a consequence of the noise-induced errors, the discrete force distributions are blurred, i.e. the force resolution is reduced. In case of white noise, the standard error 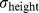 of the heights determined from the fits decreases with the square root of the fit length 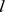:
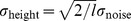
(15)


Thus, the distributions of calculated step heights are expected to be Gaussians with standard deviation 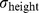. If their amplitudes are weighted by the corresponding detection rates, a prediction for the resulting histogram is possible (gray line in fig. 3b). False-positive detections cause the wrong step heights below the 5 pN peak and a reduction of 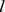 (see methods), so that the actual peaks (green) are slightly broader than predicted (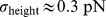). As eq. 15 is not valid for AFM noise, the resulting peaks (blue) are also wider than expected (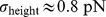). Hence, the height resolution for this kind of test signals is of the order of a pN. As a consequence of the underestimated step heights, the histogram obtained by the 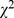 method (red) is shifted to lower forces by about 5 pN.

### Reproduction of continuous height distributions

Steps heights encountered in real data are generally not restricted to discrete values, but are continuously distributed. In practical applications, the recovery of these distributions can be highly relevant, e.g. for the analysis of force spectroscopy data. To this end, test signals of data set B with 2 to 10 steps (100 each) superimposed with AFM noise (standard deviation 10 pN) were analyzed and the step heights obtained by the 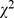 method were compared with an approach based on linear fits left and right of the step position (see methods). Noise-induced errors impair both techniques, so that the calculated heights differ significantly from the true values (fig. S5). The linear fits reproduce the continuous height distribution well for step heights above 25 pN. The 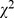 method underestimates all heights, and the shape of the resulting distribution does not resemble the actual one.

### Computational cost

Detection of steps in data with 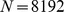 samples using a C++ implementation of the MSF algorithm with 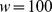 requires a computation time 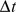 of the order of a millisecond on a current personal computer. This allows for automated processing of large data sets. 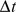 rises linearly with 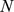. The 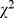 method is about 7000 times slower for the same number of samples, because it is based on more complex calculations. It performs linear fits over comparatively large intervals, partially including the same data points repeatedly [9]. The relative difference in computation time increases with 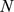 (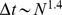).

### Analysis of AFM force spectra

**Figure 4:**
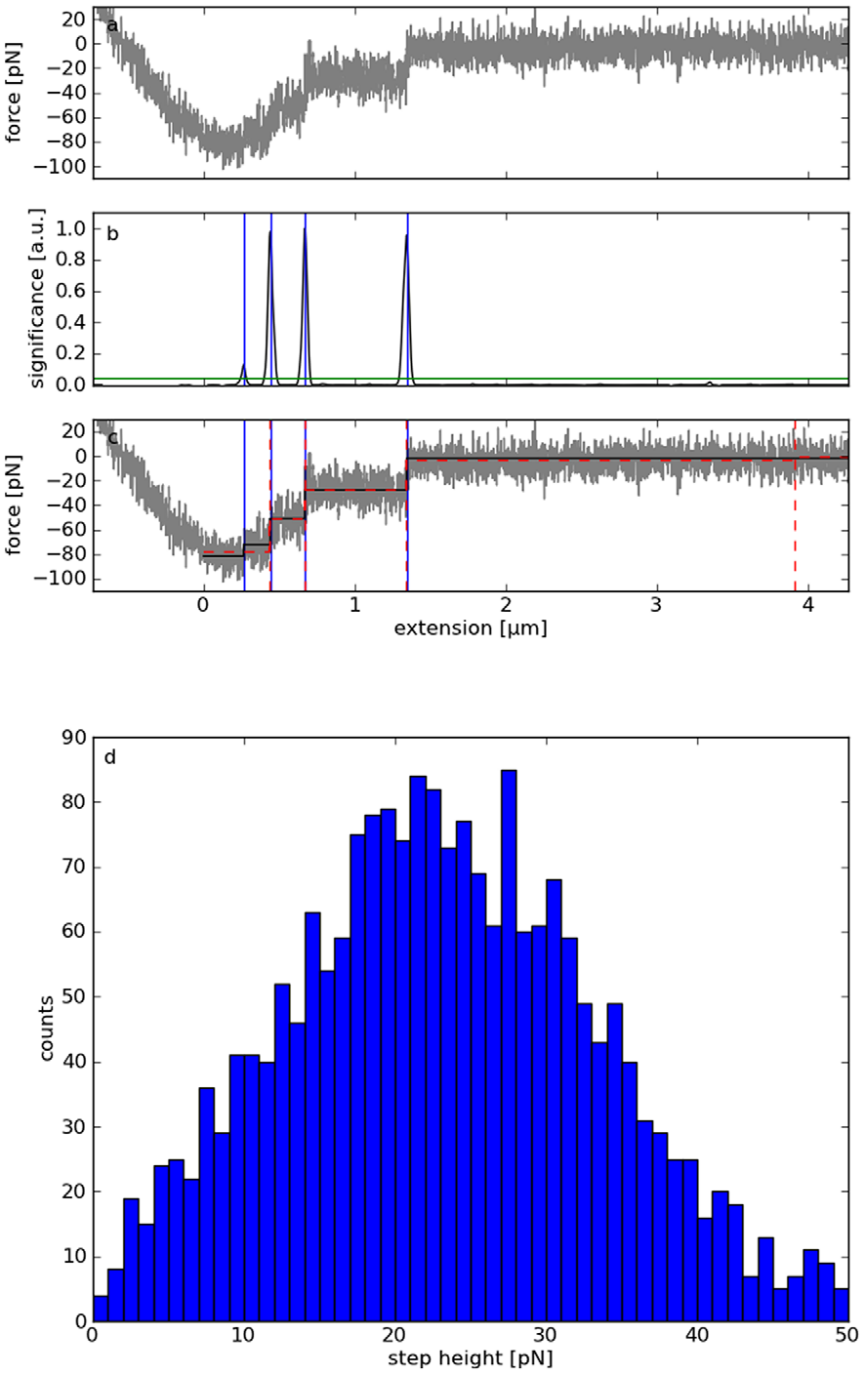
Real data measured by atomic force spectroscopy of the interaction of human T lymphocytes with the integrin VLA-4 ligand VCAM-1. a: Force-distance curve showing the typical signature of membrane tether formation. b: Local maxima of the calculated significance exceeding a threshold (green horizontal line) indicate the rupture of the tethers (blue vertical lines). c: Fitting constant plateaus piecewise to the sections between the steps yields a clean force-distance curve (black). The Kerssemakers method identifies different step positions (red). d: Distribution of the calculated step heights resulting from the analysis of about 4200 force curves by MSF.

Force-distance curves were obtained by atomic force spectroscopy measurements with membrane tethers pulled from living human T lymphocytes. The adhesion force of single tubes formed by the cell membranes when interacting with the integrin VLA-4 ligand VCAM-1 were measured as described by Schmitz et al. [14]. Bond rupture results in abrupt changes of force exerted on the cantilever. As a consequence, discrete force states are recorded (see example in fig. 4a). The steps marking the transitions between these states were detected by the MSF algorithm with manually optimized parameters (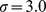, 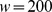) and a constant threshold for the significance of 10000 (blue vertical lines in fig. 4b and c; see methods). Both MSF parameters are higher than those resulting from the optimization based on data set B to suppress oscillations contained in the force signals, which are not modeled by the artificial AFM noise. For comparison, the Kerssemakers algorithm was also applied to the example (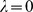). If configured to detect the same number of steps, it does not identify the first one at ≈0.25 µm with the lowest significance (red lines in fig. 4c). However, the fit indicates that it is correct.

The resulting step heights found within a maximum pulling extension of 1.5 µm show a very symmetric distribution (fig. 4). The modal of about 23 pN corresponds well to previous AFM measurements of the same cell type under comparable conditions [19]. As proven by the evaluation of simulated data, the MSF algorithm allows recovering steps, which are below the detection limit for conventional techniques (≈10 pN). In fact, the unimodal symmetric shape of the histogram provides strong evidence, that it is not substantially distorted by false-positives. An automated analysis of these data with the Kerssemakers method is not possible, because the number of steps must be specified manually for each curve.

### Analysis of kinesin motor experiments

**Figure 5:**
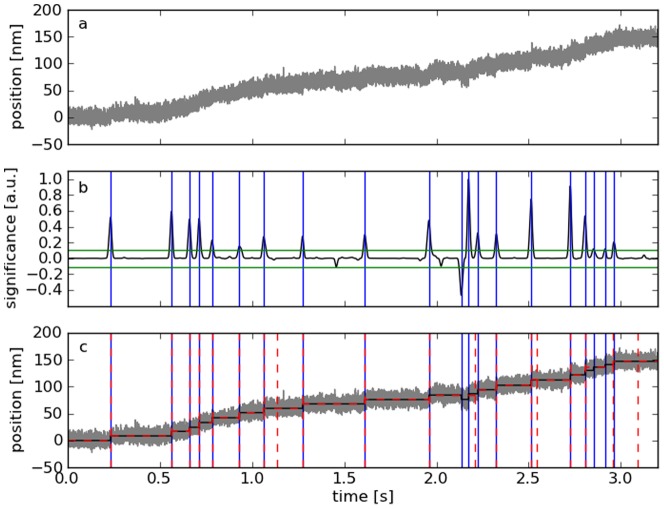
Application of the MSF method to kinesin motor data. a: Distance vs. time trace obtained by optical tweezers [20]. A polystyrene bead decorated with kinesin-2 proteins is held in an optical trap at a constant pretension of 1.4 pN, while one of the motor proteins moves along a surface-attached microtubule. b: The MSF indicator is thresholded (green) to locate the positions of the motor steps (blue). c: The mean values between these step positions were used to reconstruct the movement of the bead (black). The Kerssemakers method shows similar results, but does not detect the potential steps at ≈2.9 s, which correspond to the two lowest maxima in the MSF indicator.

Molecular motors constitute another example for a biological system showing discrete states. Both methods were applied to resolve the step-like movement of kinesin-2 along microtubules [20] with manually optimized parameters (MSF: 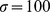, 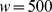, indicator threshold = 22000; Kerssemakers: 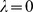, 20 steps). They show similar results, but the Kerssemakers algorithm does not detect the two potential steps at ≈2.9 s, which correspond to the two lowest maxima in the MSF indicator (fig. 5). However, the rising flank suggests that at least one of them is actually correct.

### Parameter optimization

Independently of a particular detection algorithm, steps with arbitrarily low SNRs can be recognized if they are wide enough, and narrow steps if their SNR is high enough. No simple relation exists between the method-specific parameters and the lateral or height resolution. Optimal values in terms of efficient detection of steps depend on their widths and heights (fig. S6). However, for sufficiently wide and high steps, MSF yields good results for 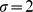 and 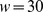, and the 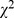 method for 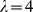. Whereas varying 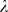 has only a moderate effect on the results, optimizing the width of the Gaussian kernel 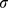 and the half window size 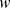 can highly improve or impair the detection efficiency, particularly for narrow steps. Ideal values of 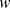 in the sense of maximum detection rates are approximately inversely proportional to the SNR for large and intermediate heights (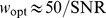). The optimal 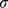 depends on 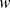 and on the step characteristics in a complex way. As a rule of thumb, a higher 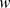 and a lower 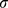 increases the height resolution, and therefore increases detection rates for low steps. If steps are lying too close together to be separated, 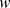 must be decreased. If false-positives appear within the flanks of the indicator peaks of correctly identified steps, 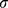 must be increased. Thereby, minor peaks with low prominences are eliminated.

The parameters can also be fine-tuned by comparing actual and detected steps in simulated signals mimicking the characteristics of real experimental data (such as noise amplitude or step heights and widths). By systematically varying the detection parameters, optimal values can be identified. If the signal characteristics are not constant, parameters resulting in maximum average detection rates can be determined by evaluating multiple test signals. This approach is illustrated using the example of single molecule force spectroscopy data obtained with living lymphocytes: The number of steps and their positions are determined by random statistic processes, which can be mimicked by Monte Carlo simulations. To create a realistic reproduction, the model parameters must be fitted to the experimental data [15]. In the next step, the model is used to create a set of artificial curves with known steps and random noise (in this example data set B). By comparing actual and detected step positions for a large batch of simulated data, optimal parameters for the simulated data can be identified. A similar procedure was performed with data set A. The optimized parameters are only valid for the chosen model. An inapplicable model results in sub-optimal parameters.

### Conclusions

An exclusion principle holds for the detection of steps: To be detected, they must be sufficiently wide if their SNR is small and their SNR must be sufficiently high if they are narrow. Within these limits, MSF can be configured to perform a long-range search for low steps or a locally confined search for narrow steps. Thereby, it generally obtains better detection rates than the 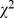 method while needing less computation time. Further, it does not require the user to specify the number of steps to be detected. Instead, a detection sensitivity can be chosen. In contrast to the Matlab implementation of Kerssemakers et al., it is able to detect single steps, decaying parts do not result in false-positive detections, and the calculated heights are correct, even if the flanks between the steps are not constant. The increased height resolution provides the possibility to detect discrete states in biological data, which are limited by low SNRs.

## Supporting Information

Figure S1 **Examples of the three types of test signals deployed for data analysis (A: constant plateaus separated by two steps of height one at a variable distance contaminated by additive white Gaussian noise, B: artificial force-distance curves mimicking single-molecule force spectroscopy experiments with living cells superimposed by AFM noise, C: like B, but exactly 4 steps at 2, 6, 10, and 14 µm with discrete heights randomly chosen from 5, 10, 20, and 40 pN).**
(PNG)

Figure S2
**Detection rates vs. noise amplitudes of the MSF and the** 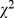 **algorithm applied to synthetic test signals for different optimization methods.** Every marker represents the average rate over 100 curves with distinct random noise, with error bars indicating the standard errors. a, c, e: Results for data set A with parameters optimized for (a) each SNR of the steps and variable width, (c) variable SNR and each width, and (e) for variable SNR and width. The last plot shows the average detection rates over 33 step distances between 40 and 1000 data points. b, d, f: Results for data set B with parameters optimized for (b) each noise level and a variable number of steps, (d) variable noise level and each number of steps, and (f) for variable noise level and number of steps. The last plot shows the average detection rates over the range of 2, 3, …, 50 steps.(TIF)

Figure S3
**True (black) and detected (blue) step positions found in data set B by MSF (a) and the** 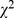 **method (b).** The numbers of false-positives (green) and false-negatives (red) are significantly lower for MSF.(TIF)

Figure S4
**True (black) and detected (blue) steps as a function of their true heights resulting from application of the MSF (a) and the** 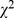 **method (b) on data set C.** The numbers of false-negatives (red) are significantly higher for the 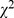 method. In contrast to MSF, it yields very few false-positives (green), but also does not reproduce the 5 pN peak at all.(TIF)

Figure S5
**True (black) and calculated (blue) step heights obtained from data set B by linear fits (a) and by the** 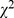 **method (b).** The former does not reproduce low steps, the latter underestimates all heights.(TIF)

Figure S6
**Influence of the SNRs and widths of steps on the detection rates and optimal parameters for (a) the size of the Gaussian kernel** 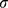 **and (b) the half width of the fit window** 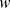 **.** Only one parameter is varied at a time, the other is held constant (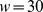, 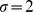). Each point represents the detection rate averaged over 100 curves of data set A with a SNR of 0.5, 1.0, or 2.0, and a step distance of 40 or 1000 data points. Optimal values for the varied parameter are marked by the circles.(TIF)
