# Supplementary figures and images for: Identifying Discrete States of a Biological System Using a Novel Step Detection Algorithm
Source: PLoS One. 2012 Nov 7;7(11):e45896. doi: 10.1371/journal.pone.0045896 (PMC3492383; doi:10.1371/journal.pone.0045896)

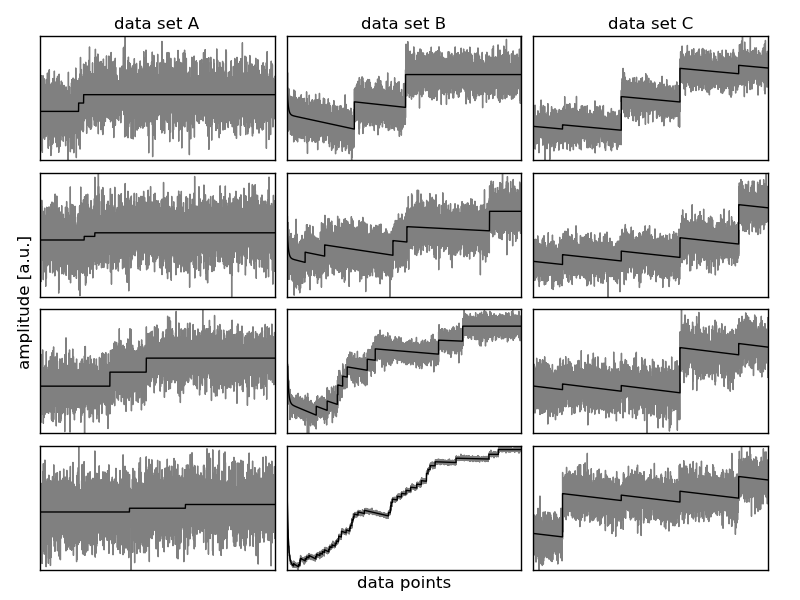

Supplement: Figure S1 — Examples of the three types of test signals deployed for data analysis (A: constant plateaus separated by two steps of height one at a variable distance contaminated by additive white Gaussian noise, B: artificial force-distance curves mimicking single-molecule force spectroscopy experiments with living cells superimposed by AFM noise, C: like B, but exactly 4 steps at 2, 6, 10, and 14 µm with discrete heights randomly chosen from 5, 10, 20, and 40 pN). (PNG) [file pone.0045896.s001.png]

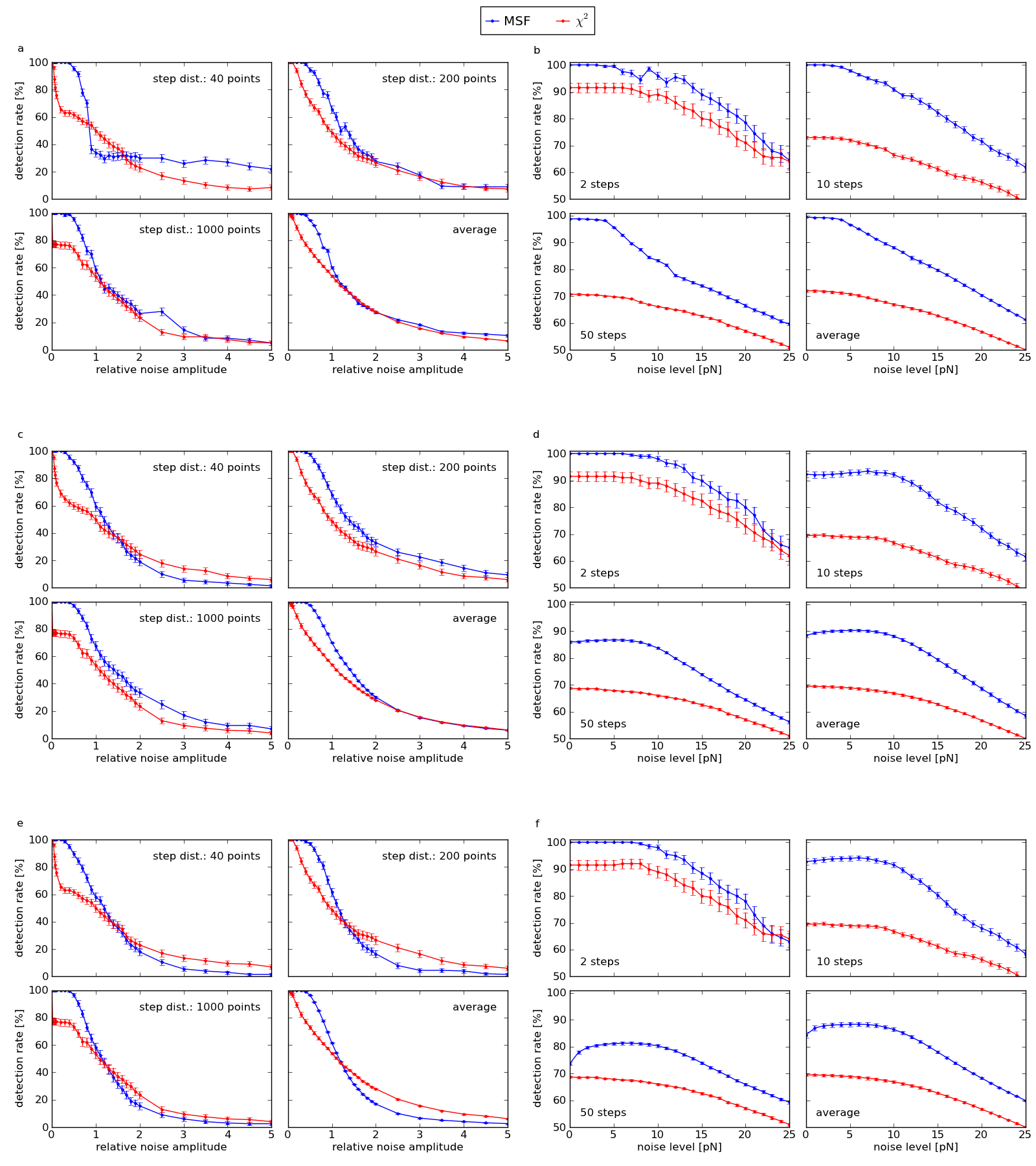

Supplement: Figure S2 — Detection rates vs. noise amplitudes of the MSF and the algorithm applied to synthetic test signals for different optimization methods. Every marker represents the average rate over 100 curves with distinct random noise, with error bars indicating the standard errors. a, c, e: Results for data set A with parameters optimized for (a) each SNR of the steps and variable width, (c) variable SNR and each width, and (e) for variable SNR and width. The last plot shows the average detection rates over 33 step distances between 40 and 1000 data points. b, d, f: Results for data set B with parameters optimized for (b) each noise level and a variable number of steps, (d) variable noise level and each number of steps, and (f) for variable noise level and number of steps. The last plot shows the average detection rates over the range of 2, 3, …, 50 steps. (TIF) [file pone.0045896.s002.tif]

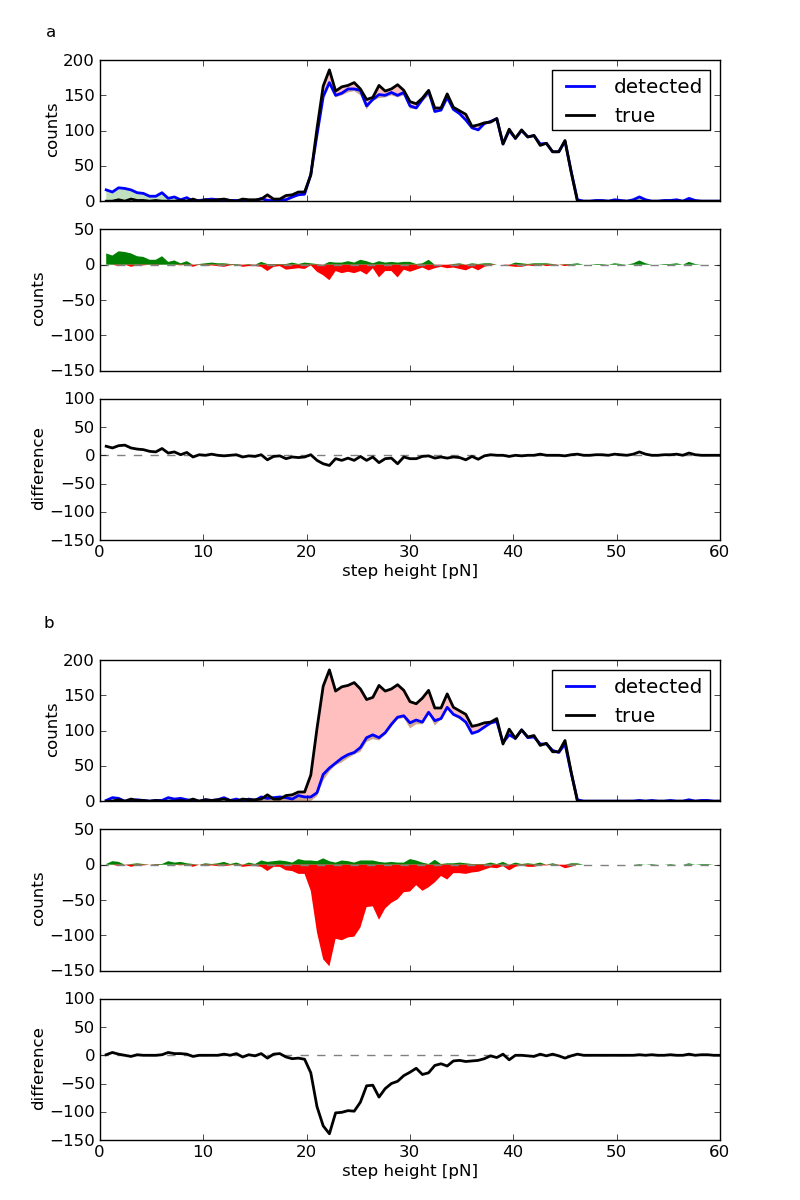

Supplement: Figure S3 — True (black) and detected (blue) step positions found in data set B by MSF (a) and the method (b). The numbers of false-positives (green) and false-negatives (red) are significantly lower for MSF. (TIF) [file pone.0045896.s003.tif]

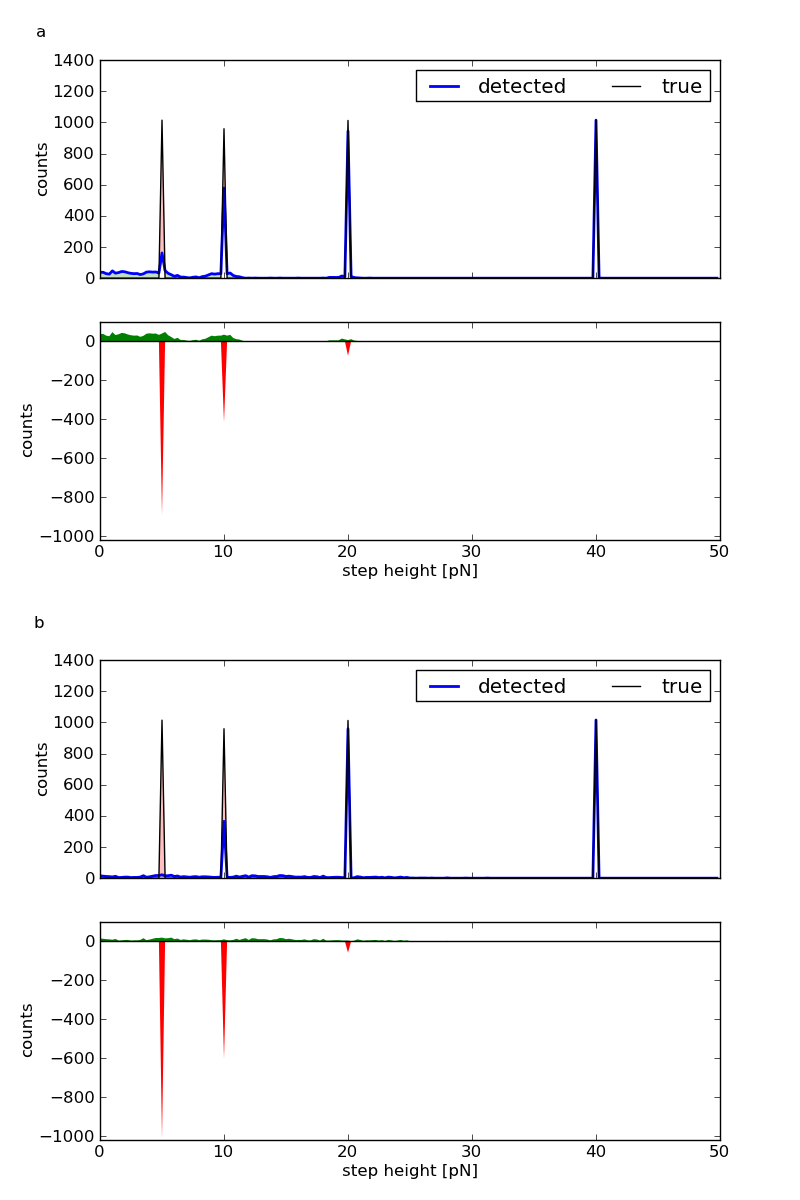

Supplement: Figure S4 — True (black) and detected (blue) steps as a function of their true heights resulting from application of the MSF (a) and the method (b) on data set C. The numbers of false-negatives (red) are significantly higher for the method. In contrast to MSF, it yields very few false-positives (green), but also does not reproduce the 5 pN peak at all. (TIF) [file pone.0045896.s004.tif]

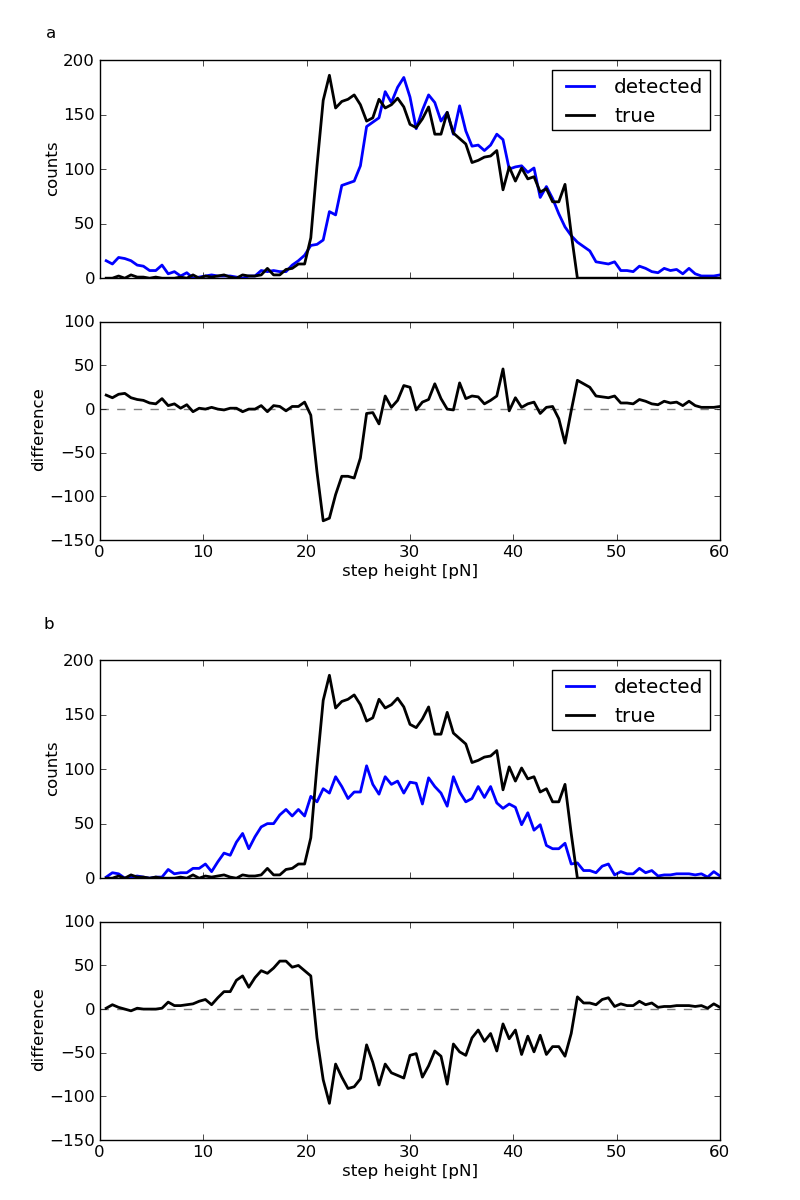

Supplement: Figure S5 — True (black) and calculated (blue) step heights obtained from data set B by linear fits (a) and by the method (b). The former does not reproduce low steps, the latter underestimates all heights. (TIF) [file pone.0045896.s005.tif]

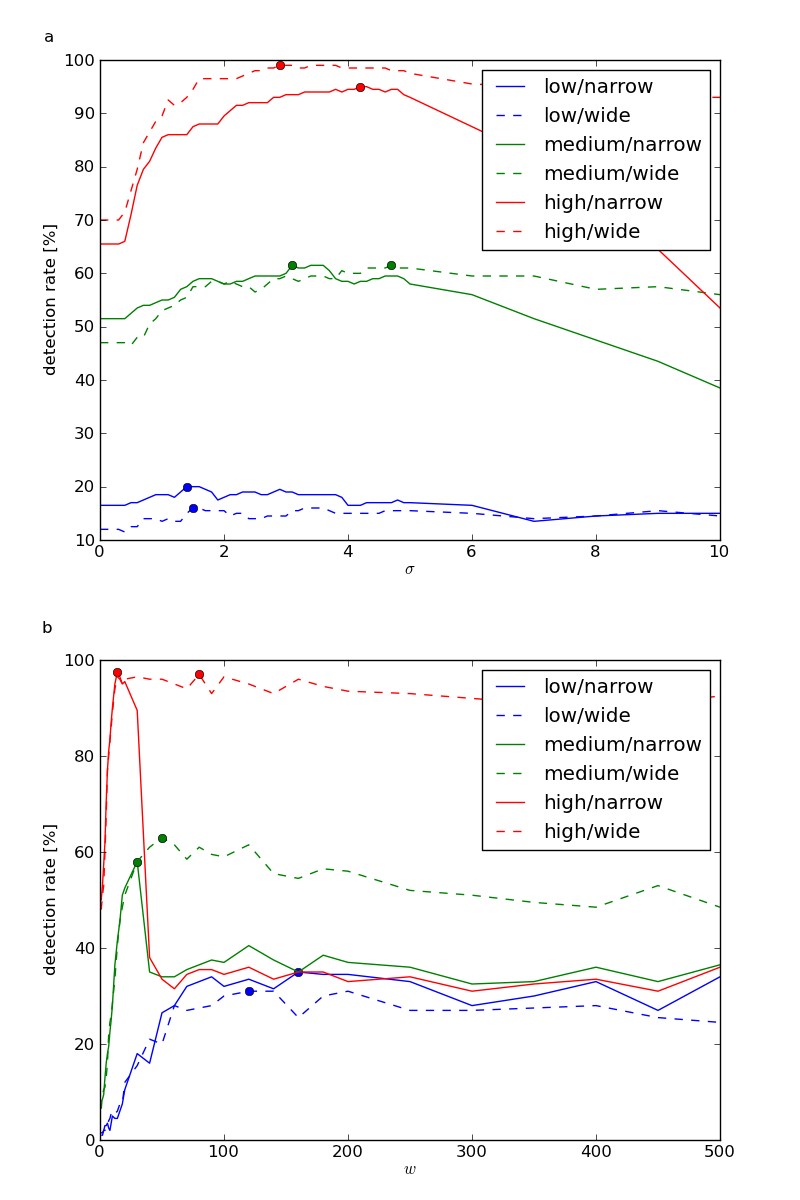

Supplement: Figure S6 — Influence of the SNRs and widths of steps on the detection rates and optimal parameters for (a) the size of the Gaussian kernel and (b) the half width of the fit window . Only one parameter is varied at a time, the other is held constant (, ). Each point represents the detection rate averaged over 100 curves of data set A with a SNR of 0.5, 1.0, or 2.0, and a step distance of 40 or 1000 data points. Optimal values for the varied parameter are marked by the circles. (TIF) [file pone.0045896.s006.tif]
